# Expression of NR2B in Cerebellar Granule Cells Specifically Facilitates Effect of Motor Training on Motor Learning

**DOI:** 10.1371/journal.pone.0001684

**Published:** 2008-02-27

**Authors:** Jianwei Jiao, Akira Nakajima, William G. M. Janssen, Vytautas P. Bindokas, Xiaoli Xiong, John H. Morrison, James R. Brorson, Ya-Ping Tang

**Affiliations:** 1 Department of Psychiatry, University of Chicago, Chicago, Illinois, United States of America; 2 Department of Neuroscience, Mount Sinai School of Medicine, New York, New York, United States of America; 3 Department of Neurobiology, Pharmacology, and Physiology, University of Chicago, Chicago, Illinois, United States of America; 4 Department of Neurology, University of Chicago, Chicago, Illinois, United States of America; Centre de Recherches su la Cognition Animale-Centre National de la Recherche Scientifique and Université Paul Sabatier, France

## Abstract

It is believed that gene/environment interaction (GEI) plays a pivotal role in the development of motor skills, which are acquired via practicing or motor training. However, the underlying molecular/neuronal mechanisms are still unclear. Here, we reported that the expression of NR2B, a subunit of NMDA receptors, in cerebellar granule cells specifically enhanced the effect of voluntary motor training on motor learning in the mouse. Moreover, this effect was characterized as motor learning-specific and developmental stage-dependent, because neither emotional/spatial memory was affected nor was the enhanced motor learning observed when the motor training was conducted starting at the age of 3 months old in these transgenic mice. These results indicate that changes in the expression of gene(s) that are involved in regulating synaptic plasticity in cerebellar granule cells may constitute a molecular basis for the cerebellum to be involved in the GEI by facilitating motor skill learning.

## Introduction

Motor learning, a process by which an animal learns to perform a motor skill more accurately and efficiently through practice, plays an essential role in human life. Unlike explicit memory such as recognition memory and spatial memory, motor learning is characterized by slow development, without the requirement of conscious recall, and in general being lifetime-lasting [1]–[3]. Based on the role of the cerebellum in motor activities such as fine motor movement and motor coordination as well as the computational network within the neural circuitries, cerebellar motor learning was first postulated by Drs. Albus and Marr [4], [5]. Since then, a huge number of studies from both clinical observations and animal researches supported this theory. However, as most of these studies were conducted in subjects with cerebellum lesion, it is unable to adequately dissociate deficits in learning associated with component movement (motor learning) from deficits in performing compound movement (such as motor coordination) [6], [7]. Following the use of selectively neurochemical approaches [8], [9], cell type-specific genetic manipulation [10], [11], and functional imaging [12], [13], it is now generally accepted that the capacity of the cerebellum to memorize motor skills is distinct from its ability to simply organize or coordinate motor activities [2], [14].

Another important component that shapes motor learning is genetic factors. Twin and adoption studies have indicated that human performance in response to exercise interventions is highly variable among individuals, which is at least in part due to variation in genes [15]. For example, polymorphisms on the gene coding for angiotension-converting enzyme significantly contribute to inter-individual variability in physical endurance performance [16]. However, the heritability for physical activities or sport participation is around 20–60% [15], [17], which is lower than 40–80% for general intelligence (40–80%) [18], [19], indicating that motor activity including motor learning is more profoundly under the influence of environmental factors. Indeed, the effects of environmental intervention on motor learning are readily measurable under certain circumstances. For example, restriction of early-life environments such as in intrauterine or at early postnatal stages may result in overall brain function decline including motor learning [20], while access to a particular motor training paradigm leads to selectively better performance in the task that used in this program [21], [22].

Which factor, genetic or environmental, is relatively more important for motor learning is a longstanding debate regarding the influence of nature vs. nurture in human behaviors [23]. Evidence from longitudinal and cross-sectional studies suggests that in most cases gene/environment interaction (GEI) plays an essential role in constraining various human behaviors including cognition, personality, social activity, and personal interests such as sport participation [24]–[26]. Most interestingly, several lines of evidence clearly show the role of GEI in the development of motor learning. For example, in a human rotary pursuit task, a task that is relatively unaffected by cognition [27], the performance is driven by an integrative influence from both heritability and practice [28]. Similar interactions are also observed in other motor learning paradigms [29], 30. In addition, the development of super motor skills requires environmental interventions at early critical stages of life, together with many other psychological factors [31]. Taken together, these studies suggest that GEI represents an integrative driving-force to constrain the development of motor skills.

However, the molecular basis that underlies the effect of GEI on motor learning is still far from clear. It is well accepted that motor learning undergoes a typical form of use-dependent plasticity in the brain [21], [32]. At the same time, it has been well established that the NMDA receptor (NMDAR) plays a central role in synaptic plasticity [33]. Accumulating evidence has indicated that NMDAR also plays a role in motor learning [34]–[36]. It should be noted that different assemblies of NMDAR complex has different channel properties [37]. Our previous work in the hippocampus of the mouse demonstrated that recombinant NMDAR with NR2B overexpression enhanced both synaptic plasticity in the hippocampus and hippocampal-dependent learning [38]. Most interestingly, recent evidence has revealed a critical stage (1–1.5 months) for an enhanced synaptic plasticity, and this enhancement is dependent on NR2B containing NMDAR [39]. Therefore, it is of significant interest to determine whether recombinant NMDAR changes cerebellar-learning.

## Results

### Generation of inducible cerebellar granule cell-specific NR2B transgenic mice

**Figure 1:**
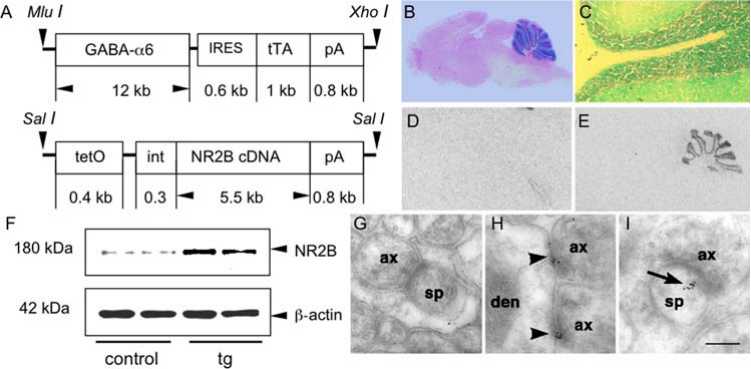
Inducible cerebellar granule cell-specific NR2B transgenic mice. A. Expression vectors for GABA-α6-tTA (upper panel) and tetO-NR2B (low panel). pA: poly-A signal; int: artificial intron. B and C. Granule cell specific expression. LacZ staining reveals that blue stain is exclusively observed in the cerebellar cortex (B) and is restricted to granule cells only (C). D and E. Distribution of the NR2B transgene in the cerebellum. *in situ* hybridization shows that the expression of the NR2B transgene is exclusively observed in the cerebellar cortex of tg (E), but not of control mice (D). F. Expression level of the NR2B transgene. Western blot shows the expression of NR2B protein (180 kDa) in the cerebella of tg mice, but not of control (cont) mice. ß-actin blot (42 kDa) shows the relative amount of protein loading. G–I. Synaptic localization of NR2B protein. EM immunocytochemistry shows positive gold-particles in many mossy fiber/granule cell synapses of tg (H and I), but not of control mice (G). The immunoreactivity in tg mice is associated within the “active zone” (postsynaptic density and synaptic cleft, arrow heads) of the synapse (H; arrowhead) or is associated throughout the spinous cytoplasmn and neuropil of spine head (I; arrow). ax: axon; den: dendrite; sp: spine. Scale bars in I = 0.3 µm.

The most unique feature for NMDAR in the cerebellum is that the expression of NR2 subunits is fundamentally different in different cell types across different developmental stages [40]. In granule cells, NR2B is expressed during the early postnatal stage and is replaced by NR2A and then by NR2C so that the dominant type of the NMDAR in adult stages is NR1/NR2C complex [40]. This development-dependent switching provides an ideal model to study how gene expression affects cerebellar function. Accordingly, we generated inducible cerebellar granule cell-specific NR2B transgenic mice. As shown in Figure 1A, two single transgenic mouse strains were generated. A granule cell-specific promoter, GABA-α6 promoter [41], was used to drive tTA . In order to confirm the specificity and efficacy of this promoter, we first generated *GABA-α6-*tau-LacZ reporter mice. As shown in Figure 1B and C, Lac-Z staining revealed that blue stain was only observed in the cerebellar cortex and was restricted to granule cells including soma (granular layer), dendrites, and parallel fibers throughout the molecular layer in transgenic (Figure 1B and C), but not wild-type mice (data not shown). Then *GABA-α6-tTA/tetO-NR2B double transgenic* (simply tg hereafter) mice were produced and these mice showed normal growth, eating behavior, body weight, and breeding behaviors (data not shown). *in situ* hybridization revealed that the expression of NR2B transgene was exclusively observed in the cerebellar cortex of tg mice (Figure 1E), but not of their littermate control (thereafter control) mice (Figure 1D). Western blot confirmed the expression of NR2B protein in the cerebella of tg mice (Figure 1F). Unlike AMPA receptor, trafficking of NMDAR, especially for NR2B subunits, does not essentially or necessarily require other partners [42]. Therefore, it was expected that the expression of tg NR2B protein was able to move to the synapses. However, it was recently reported that in the cultured hippocampal neurons, the increased NR2B expression did not increase the synaptic NR2B-containing NMDAR [43]. To address whether there is a difference between *in vitro* and *in vivo* systems, we used electron microscopy (EM) immunocytochemistry to confirm NR2B synaptic expression. As shown in Figure 1G–I, axo-spinous and axo-dendritic synapses throughout all groups were morphologically intact. Immuno-gold particles, which represent NR2B immunoreactivity, were found in mossy fiber/granule cell synapses of tg (Figure 1H and I), but not control cerebellum (Figure 1G). In these labelled synapses, gold particles were closely associated with the “active zone” (PSD and synaptic cleft) of synapses (Figure 1 H). In addition, some immunogold-labelled synapses expressed NR2B throughout the active zone and neuropil of the spine head (Figure 1I). Qualitative analysis indicates a dramatically increased NR2B level (p<0.001, Mann-Whitney *U* test) in tg mice [4.3 (mean)±2.4 (SD); n = 5], but not control mice [0.8±0.7; n = 5], indicating that the products of NR2B transgene are able to transport into the synapses, which is different from *in vitro* condition [43]. All mice used here were around 2–3 months old, with mixed sexes.

### NR2B transgene in cerebellar granule cells of tg mice is functional

**Figure 2:**
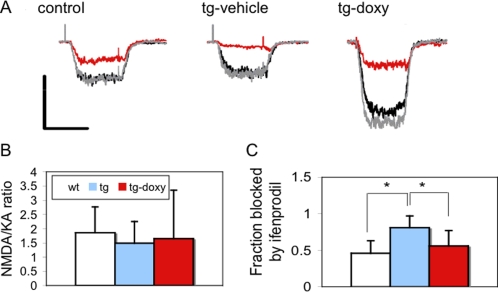
NMDA currents in recombinant NMDAR in cultured granule cells from tg mice. A. Representative current traces elicited by NMDA (300 µM) alone (black lines), NMDA with 10 µM ifenprodil (red line), and after ifenprodil washout (gray line). Application of NMDA with 10 µM glycine in Mg^2+^-free solution triggered robust NMDA-current in control and tg cells, while no significant difference in current peak or decay time was found between them. Vertical scale bar, 150 pA for control and tg cells, 300 pA for tg cells-treated with doxy; horizontal scale bar, 400 msec. B. Whole-cell responses to NMDA normalized by kainate were not significantly different between control and tg cells. C. Fractional block of NMDA responses by ifenprodil. Application of ifenprodil partially blocked the current in control cells and almost completely blocked the current in tg cells. When tg cells were treated with doxycycline, the blocking effect of ifenprodil returned to the level of control cells. *: p<0.05, one-way ANOVA, followed by *Fisher's PLSD* test. Data are expressed as mean±SD.

To determine whether the expressed NR2B is functional, we characterized ligand-gated currents mediated by NMDA in cultured granule cells from cerebella of control and tg pups at P8, by using whole-cell patch-clamping recordings. No significant differences in current peak, decay time, or ratio of NMDA over kainate-evoked currents were found between control and tg cells (Figure 2A and B). This might be due to the reason that the transgene expression was not fully triggered at 13–18 days *in vitro* culture from P8 pups, since the GABA-α6 promoter starts to function at about P14 *in vivo*. In addition, the use of ifenprodil, a specific NR2B antagonist, revealed a significant NR2B component in control cells, indicating that the endogenous NR2B expression at the early developmental stage may also buffer the changes that are associated with the transgene expression. However, our results also revealed a significantly higher sensitivity of tg cells (treated with vehicle) to ifenprodil, compared to either control cells-treated with vehicle (p<0.05) or tg cells-treated with doxy (p<0.05) (Figure 2C), indicating that transgene is potentially functional.

**Figure 3:**
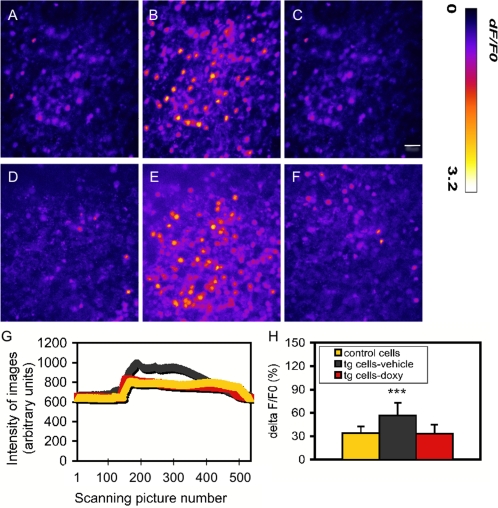
Enhanced NMDAR function in cerebellar granule cells of tg mice. A–F. Representative Ca^2+^ imaging microphotos from before the NMDA (0.1 µM together with glycine 10 µM) application (A and D), the maximal [Ca^2+^]_I_ responses (B and E), and returned to the basal level (C and F) in control cell (A–C) and tg cell (D–F), respectively. G. Representative time courses for the changes of [Ca^2+^]_i_ in single granule cells of three groups of mice tested during a typical 5-minute experimental period, during which 500 image frames were collected. H. Quantitative analysis of [Ca ^2+^]_i_. Data is expressed as mean±SD. ***, p<0.001, *post-hoc* analysis with *Fisher's PLSD* test in comparison between tg neurons-treated with vehicle and control neurons and between tg neurons-treated with vehicle and tg neurons-treated with doxy, respectively. Bar in F represents 20 µm. Data are expressed as mean±SD.

In order to solve the developmental concern described above, we further examined NMDA-evoked Ca^2+^ influx in granule cells in cerebellar slices prepared from mice at the age of 30 days, during which the expression of the transgene was fully expressed under the control of the GABA-α6 promoter. As shown in Figure 3A–F, the application of NMDA triggered robust intracellular Ca^2+^ influx ([Ca^2+^]_i_) in both control (Figure 3A–C) and tg cells (Figure 3D–F). Figure 3G shows the representative curves for the changes in intensity of fluorescence signal in a single granule cell from three groups of mice tested. Quantitative analysis of [Ca ^2+^]_i_ indicated that the basal [Ca ^2+^]_i_ in tg neurons was 147 nM±10 (mean±SD), which was similar to 137 nM±9 in control neurons. After stimulation with 0.1 µM NMDA, however, the [Ca ^2+^]_ i_ in tg neurons (397 nM±90) was significantly higher than in control neurons (261 nM±46). [Ca ^2+^]_ i_ was calculated based on the equation: [Ca ^2+^]_i_ = Kd(F-F_minimum_)/(F_maximum_-F) (*28*). Data were collected from a randomly selected region from each cerebellar slice and at least 10 neurons were recorded in this region. A total of 4 slices were examined in each mouse and there were 5 mice in each group. One-way ANOVA revealed a highly significant difference in Δ F/F_0_ among these three groups [F(2,58) = 22.16, p<0.001] and *post-hoc* analysis revealed a highly significant difference between tg neurons-treated with vehicle and control neurons-treated with vehicle (p>0.001) and between tg neurons-treated with vehicle and tg neurons-treated with doxycycline (p>0.001) (Figure 3H). These results demonstrated that the functional significance of the transgene is more evident in the older stage than in the early developmental stages.

### Expression of NR2B in cerebellar granule cells does not result in observable morphological changes in the cerebellum of tg mice

**Figure 4:**
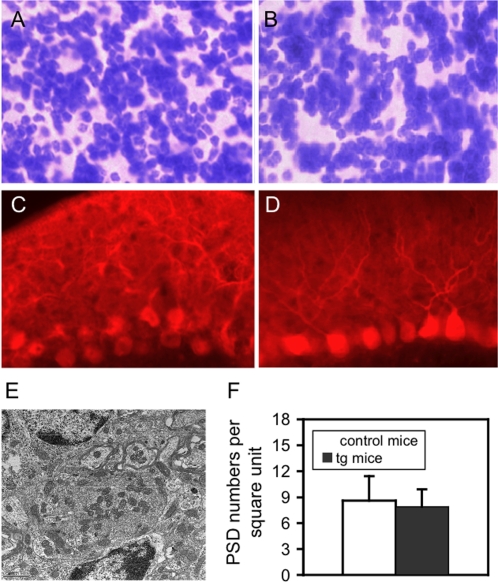
Morphological changes in the cerebellum of tg mice. A and B. Nissl straining of sagital sections from control (A) and tg (B) mice. No observable difference, either in general morphology of granule cells or cell number in granule cell layer, was found between them. C and D. Immunohistological staining with DK-28 (Sigma). No obviously morphological changes could be found in the Purkinje cell layer and the molecular cell layers between control (C) and tg mice (D). E. A typical glomerulus from cerebellar section from a tg mouse. The bar in left corner was 1 µm. F. Quantitative analysis of number of mossy fiber/granule cell synapses. No significant difference was found between control and tg mice. Data are expressed as mean±SD.

As higher Ca^2+^ influx following the activation of NMDAR was observed in tg slices, we carefully examined the morphological changes at four levels. At the gross level, we examined cerebellum size, weight, and structural organization and there was no observable difference between control and tg mice (data not shown). At the microscopic level, we examined whether there was any obviously morphological changes in tg mice with Nissl straining (cresyl violet). The results showed no observable difference, either in general morphology or granule cell number in granule cell layer between control (Figure 4A) and tg (Figure 4B) mice. It should be noted that inputs from both parallel fibers and climbing fibers to Purkinje cells form excitatory synapses and that each Purkinje cell receives input from as many as million granule cells. It might be possible that the enhanced NMDAR function in granule cells may affect survival or morphology of Purkinje cells. Accordingly, at the immunohistological level, we performed Purkinje cell-specific immunostaining with DK-28 (Sigma). No obviously morphological changes could be found in the Purkinje cell layer and the molecular cell layers between control (Figure 4C) and tg mice (Figure 4D), where Purkinje cell dendrites are observable. In addition, Purkinje cell axons can be clearly observed in the granule cell layer (data not shown). Finally, at the ultrastructural level, we used EM to determine whether the expression of NR2B affects mossy fiber synapses onto granule cells. Figure 4E shows a typical glomerulus (mossy fiber synapse) and Figure 4F indicates no significant difference (Mann-Whitney *U* test) between control and tg mice. All these results indicate that there are no observable morphological changes in the cerebellum of our transgenic mice.

### Expression of NR2B in cerebellar granule cells does not significantly change motor functions in tg mice under the standard housing condition

**Figure 5:**
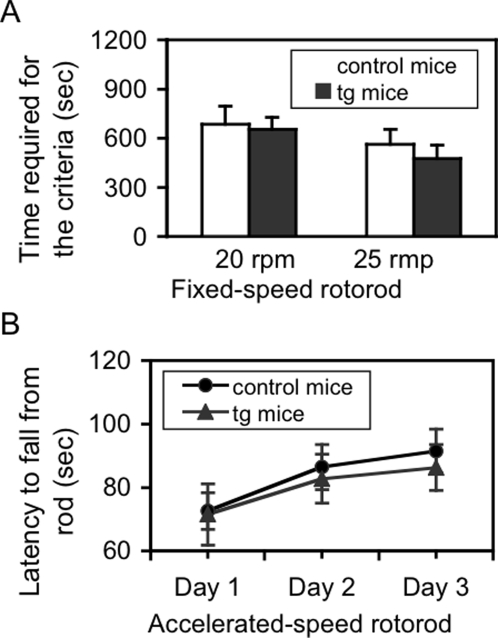
Effects of the NR2B transgene on motor learning under the standard housing condition. A. Motor learning in fixed-speed rotorod test. Two speeds, 20 rpm and 25 rpm, were used. Although a tendency of less total time required to reach the learned criterion is shown in tg mice (n = 8), this tendency does not reach statistical significance, when compared to control mice (n = 11) either in 20 rpm or 25 rpm fixed-speed test. B. Motor learning in an accelerated-speed rotorod test. The performance in both control (n = 10) and tg (n = 9) mice is better following trials and one-way ANOVA analysis reveals a significant difference in either control [F(2,27) = 9.98, p<0.001] or tg mice [F(2,24) = 10.23, p<0.001]. However, no significant difference in either the learning curve or in each training session can be found between control and tg mice. Two different sets of mice were respectively used for fixed- and accelerated-speed rotorod tests. Data are expressed as mean±SD.

We hypothesized that the enhanced NMDAR function in granule cells would lead to changes in motor activity. We first examined the general motor activity in an open-field test. Both horizontal and vertical behaviors were similar between control and tg mice (data not shown). We then employed two versions of rotorod task to evaluate motor learning. In a fixed-speed version, no significant difference in the time required to reach the criterion for having learned the task was found between control and tg mice (Figure 5A). In an accelerated-speed version, both control and tg mice learned the task following the trials, and there was no significant difference in either learning curve or each training session between control and tg mice (Figure 5B), indicating that the expression of the NR2B transgene only in the cerebellum could not produce an observable change, either enhancement or impairment, in motor activity or motor learning. Mice used here were 2–3 months old, with mixed sexes.

### Voluntary motor training (VMT) significantly enhances motor learning in the mouse

**Figure 6:**
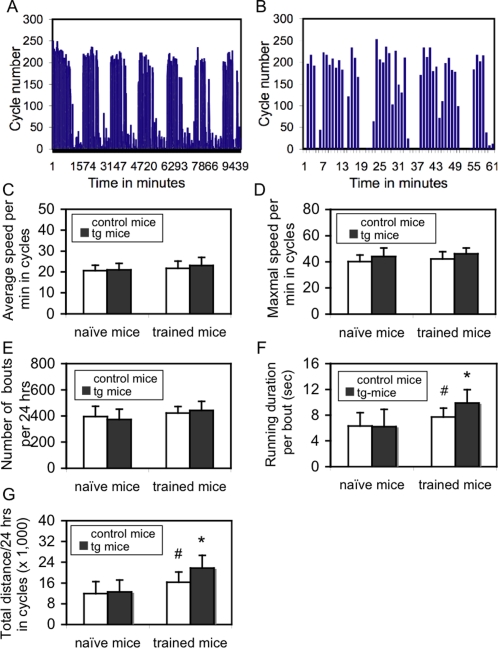
Effects of VMT on running behaviors. A. A typical circadian running behavior in a 1-week period from a tg mouse. Two circadian phases are clearly shown within every 24 hrs. Running phase was generally from 19:00 PM to 07:00 AM and non-running phase was from 07:00 AM to 19:00 PM. A low running activity was still observed in both control and tg mice during the non-running-phases. No any significant difference in this circadian pattern was found between control and tg mice. B. A typical running curve in a 1 hr-duration during a running phase from a tg mouse. C. Average running speed in cycles per minute. There was no significant difference between naïve control and naïve tg mice, between trained control and trained tg mice, and between trained and naïve mice. D. Maximal running speed in cycles per minute. There is no significant difference between naïve control and naïve tg mice, between trained control and trained tg mice, and between trained and naïve mice. E. Number of cycling bouts per 24 hrs. There was no significant difference between naïve control and naïve tg mice, between trained control and trained tg mice, and between trained and naïve mice. F. Running duration per bout. There was no significant difference between naïve control and naïve tg mice. However, a significant prolonged duration was observed in trained control mice, when compared to naïve control mice (#, p<0.05, Student's *t* test), and in trained tg mice, when compared to either trained control mice (*, p<0.05, Student's *t* test) or naïve tg mice (*, p<0.05, Student's *t* test). G. Total running distance in cycles per 24 hrs. There is no significant difference between naïve control and naïve tg mice. Similar to running duration, there was a significant difference in total running distance between naïve control and trained tg mice (#, p<0.05, Student's *t* test), between trained control and trained tg mice (*, p<0.05, Student's *t* test), between naïve tg and trained tg mice. Data are expressed as mean±SD.

Environmental intervention may significantly shape or constrain motor learning [44], [45]. We then examined whether a specific motor training would specifically enhance motor learning. As both task-specificity and slow-development are most essential features for the development of motor learning, an ideal motor training paradigm should be long-term and voluntary, and display measurable effect with a task. Mice voluntarily run in a wheel (wheeling) and the motor mechanism for wheeling is similar to the rotorod task. Accordingly, we designed a specific VMT paradigm. Both control and tg mice were subjected to VMT for 60 days from weaning (P20) up to P80. In order to avoid mouse plug during VMT, only male mice were used. We first examined the effect of the transgene and VMT on wheeling behaviors by using a homecage activity-scanning system for one week. This system offers an automatically digitized-24-hr real-time analysis of wheeling behaviors including running times or bouts (times a mouse gets up to the wheel to run at least for 2.5 cycles), running time (duration) in each bout, total running distance (in cycles), and average/maximal running speed (in cycles per minute) on a single mouse/single cage basis. Four groups of mice, both control and tg mice with VMT (trained) and without VMT (naïve), were examined. Figure 6A and B respectively show a typical circadian pattern of running activity in a one-week period and a running curve over a 1 hr-duration in a running phase from a tg mouse. The circadian pattern was the same between control and tg mice (data not shown). There was no significant difference in the average (Figure 6C) or maximal running speed (Figure 6D), numbers of bouts (Figure 6E) between naïve control and naïve tg mice, between trained-control and trained-tg mice, or between naïve and trained-mice. However, VMT significantly prolonged duration (Figure 6F) and total distance (Figure 6G) in either control (p<0.05) or tg mice (p<0.01), compared to naïve control and naïve tg mice, respectively. Interestingly, significant differences in both the duration (Figure 6F) and total travel distance (Figure 6G) were also observed between trained-control and trained-tg mice (p<0.05, respectively). Mice used here were around 80 days old. These results indicate that VMT, as an environmental intervention, is able to significantly enhance certain running behaviors and that the expression of the NR2B transgene in the cerebellum significantly strengthens this effect.

**Figure 7:**
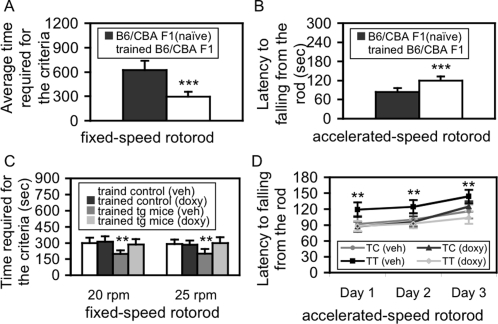
Interaction between NR2B expression and VMT in motor learning. A. VMT significantly enhances motor learning in fixed-speed rotorod in B6/CBA F1 mice. The average time required to reach the learned criterion from both 20 rpm and 25 rpm tests was significantly decreased (p<0.001, Students *t* test) in trained B6/CBA F1 mice (n = 9), compared to naïve B6/CBA F1 mice (n = 12). B. VMT significantly enhances motor learning in accelerated-speed rotorod in B6/CBA F1 mice. The average latency to falling down from the rod from 9 trials was significantly increased (p<0.001, Students *t* test) in trained B6/CBA F1 mice (n = 9), in comparison to naïve B6/CBA F1 mice (n = 10). C. The effect of GEI on motor learning in fixed-speed rotorod. Four groups of mice, trained control-vehicle (n = 12), trained control-doxy (n = 8), trained tg-vehicle (n = 10), and trained tg mice-treated with doxy (n = 12) were examined with two fixed-speed rotorod tests at 20 rpm and 25 rpm, respectively. A significant difference was observed between trained tg-vehicle mice and either trained control-vehicle or trained control-doxy, but not between trained control-vehicle and trained control-doxy, in either 20 rpm or 25 rpm tests (p<0.01, Students *t* test, respectively). When trained tg mice were treated with doxy, the performance was returned to the level of trained-control mice. D. The effect of GEI on motor learning in accelerated-speed rotorod. Four groups of mice, trained control-vehicle (n = 12), trained control-doxy (n = 11), trained tg-vehicle (n = 12), and trained tg mice-treated with doxy (n = 9) were examined with accelerated-speed rotorod. In addition to a highly significant difference in trained-tg-vehicle mice over other groups, *post-hoc* analysis with *Fisher's PLSD* test revealed a significant difference between trained tg-vehicle mice and any group of the three control groups (p<0.01, respectively). Data are expressed as mean±SD. TC: trained control; TT: trained tg.

Running in a wheel has a similar motor mechanism to performing in the rotorod task, and this concept was exemplified when we examine how VMT changed motor learning in B6/CBA F1 mice (2–3 months old; Jackson Laboratory) with two versions of rotorod task. After VMT, average time required to reaching the learned criterion in fixed-speed version (Figure 7A) and average latency to falling off the rod of 9 trials in accelerated-speed version (Figure 7B) were significantly (p<0.001) decreased and increased, respectively. In order to detect whether the effect was specifically due to wheeling, another group of B6/CBA F1 mice was subjected to modified environmental enrichment, which was the same as in our previous publication [46], except for the absence of a wheel, for the same period of time. Motor learning was examined by the same rotorod. The results did not reveal any significant effect of this wheel-absent “environmental enrichment” on motor learning (data not shown). All these results established the effect of the VMT on rotorod motor learning in the moue.

### GEI between NR2B expression and VMT significantly enhances motor learning

To test whether there was a GEI in our tg mice, four groups of mice were subjected to VMT for 2 months from weaning (P20) to P80. Two groups (e.g. control and tg mice) were treated with vehicle and the other two groups were treated with doxy (2 mg/100 ml) in dinking water from the start of VMT (P20) to the completion of rotorod tasking. Interestingly, after VMT, trained-tg-vehicle mice showed significantly enhanced performances in both 20 rpm and 25 rpm fixed-speed versions, compared to trained-control-vehicle, trained-control-doxy, and trained-tg-doxy mice (p<0.01, respectively; Figure 7C). In addition, the performance in trained-tg-doxy mice was undistinguishable to the trained-control-vehicle or trained-control-doxy mice (Figure 7C), indicating that the enhanced effect is due to the transgene expression. In the accelerated-speed version with another set of mice, similar results were observed (Figure 7D). One-way ANOVA revealed a highly significant difference among these groups [F(3,40) = 24.72, p<0.001]. *Post-hoc* analyses showed a significant difference between trained-tg-vehicle and either trained-control-vehicle or trained-control-doxy mice in each training session (p<0.01, respectively) and between trained-tg-vehicle and trained-tg-doxy mice in each training session (p<0.01, respectively). These results indicate that the expression of the NR2B transgene in the cerebellar granule cells significantly facilitates the effects of VMT on motor learning.

### The effect of GEI on motor learning is age-dependent

**Figure 8:**
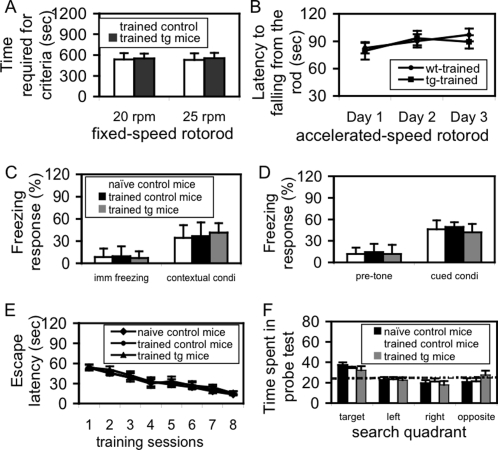
GEI is age-dependent and motor learning-specific. A. Motor learning in fixed-speed rotorod. Mice received VMT from 3 months old up to 5 months old. No significant difference was observed in either speed between trained control (n = 8) and trained tg mice (n = 11). B. Motor learning in accelerated-speed rotorod. Mice received VMT from 3 months old up to 5 months old. No significant difference was observed in either learning curve or each training session between trained control (n = 10) and trained tg mice (n = 9). C and D. Fear-conditioning. Neither two-way AVONA (transgene×VMT) nor the followed *post-hoc* analyses revealed a significant difference in freezing response in either the contextual conditioning (C) or cued conditioning (D) among the three groups of naïve control (n = 10), trained control (n = 7) and trained tg mice (n = 7). Imm: immediate; Condi: conditioning. E and F. Water maze test. Cross-sectional analysis of escape latency with one-way ANOVA indicated a highly significant learning improvement following trials in naïve control [F(5,54) = 6.412, p<0.001; n = 10), trained control [F(5,54) = 7.012, p<0.001; n = 10), and trained tg mice [F(5,60) = 7.908, p<0.001; n = 11)]. However, group comparison with the same two-way AVONA and *post-hoc* analyses did not reveal any significant difference in escape latency during the training sessions (E). Student's *t* test did not reveal a significant difference in the probe test between any two groups (F).

To test whether the GEI was affected by aging, both control and tg mice were subjected to the same VMT described above, but the training began at the age of 3 months old, in contrast to at P20 above. Motor learning was examined with fixed-speed and accelerated-speed rotorod tests. No significant difference could be found between trained-control and trained-tg mice in either fixed-speed rotorod (Figure 8A) or accelerated-speed rotorod (Figure 8B), indicating that the effect of GEI on the development of motor learning is age-dependent. Furthermore, an integrative analysis with naïve control mice indicated that although a tendency to decrease the time required to reaching the criteria in fixed-speed rotorod and a tendency to increase the latency to falling from the rod in accelerated-speed rotorod was observed in trained-mice in comparison to naïve mice, no significant difference was observed, indicating that the effects of the VMT on motor learning is also age-dependent. All these results suggest that a critical time-window exists between P20 and P80 for the development of super motor learning that is associated with this specific GEI.

### The effect of GEI is motor learning-specific

In order to determine whether the GEI affected other memory functions, we examined emotional and spatial memory function in three groups of mice (naïve-control, trained-control, and trained-tg). In fear-conditioning test, there was no significant difference in either contextual (Figure 8C) or cued conditioning freezing (Figure 8D). In a water-maze test, no significant difference in either escape latency during training session (Figure 8E) or time spent in the target quadrant in a probe test (Figure 8F) was found among these three groups. These results indicated that the effect of VMT is motor learning-specific and neither the expression of NR2B in the cerebellum nor the VMT significantly affects the emotional/spatial memories.

## Discussion

In this study, we validated a specific GEI model in facilitating the development of motor skill learning in the mouse and provided valuable insight regarding molecular/neuronal basis for motor learning from three aspects. Firstly, at the neuroanatomical level, we demonstrated that the cerebellum is a critical brain organ for this GEI. Despite that GEI in motor learning has been extensively studied [17], [28]–[30], the neuroanatomical basis for the brain involved in GEI is still elusive. In this study, we first demonstrated that the expression of the NR2B transgene in cerebellar granule cells in our transgenic mice was functional (Figure 2 and Figure 3). It has been suggested that the majority of input information in the cerebellum is transmitted from mossy fibers to parallel fibers (granule cells) and then to Purkinje cells [47]. Based on the Marr-Albus motor learning model, input from mossy fibers encodes learning information in terms of contexts and stimulation while input from climbing fibers provides either positively or negatively instructive signals to modify the efficacy of the input from parallel fibers to Purkinje cells [48], [49]. Hence, a functional change in granule cells may directly affect the information integration within the cerebellar computational network and subsequently affect motor learning. Although robust evidence is available from various lesion studies showing that lesion to granule cells impaired motor learning [2], [49], [50], our study has for the first time demonstrated that change in NMDAR function in granule cells constitutes a molecular basis for the involvement of the cerebellum in GEI in facilitating the development of better motor skill learning.

Secondly, at the system level, we found that the environmental factors may play a dominant role in the development of motor skills. Based on the different channel properties such as NR1/NR2B receptors having a longer decay time in comparison to NR1/NR2C [37], our previous study [38] together with many others [51]–[54] have established that overexpression or up-regulation of NR2B in the forebrain of animals specifically facilitates synaptic plasticity and hippocampus-dependent memory. However, in the present study, the expression of NR2B transgene only did not produce any observable changes in motor learning, indicating a fundamental difference in the process to encode cerebellar memory vs. to encode hippocampal memory in the brain. For hippocampal memory, an existing mechanism that is favourable for memory formation such as an enhanced NMDAR function is critical and once the memory trace is consolidated, this mechanism is no longer required for the retrieval [55]. Therefore, the acquisition and consolidation processes are distinctive. The consolidating process may be not necessarily nor essentially dependent on times or duration of a memorable episode presented [56]. For motor learning, however, a long-duration of neuronal activity-triggered by motor activity or motor training is required [2]. The acquisition and consolidation processes essentially overlap and the memory trace is established based on a use-dependent manner that is correlatively related to the duration or times of motor training [7]. Therefore, motor activities/training, or environmental factors, have a higher impact in the development of motor skill learning.

Thirdly, from the synaptic plasticity angel, we demonstrated that the recombinant NMDAR in cerebellar granule cells facilitates the development of this use-dependent plasticity in our transgenic mice. We first established that the expression of NR2B has an effect on long-term VMT, which was evidenced by increased running duration/distance without change in running bouts in wheeling after VMT (Figure 6). The increases in running duration/distance indicate that wheeling skill may be enhanced, as running duration/distance gradually increases following wheeling (data not shown). If this enhanced “wheeling skill” is associated with some kind of use-dependent plasticity, the expression of NR2B facilitates the development of this kind of plasticity. Indeed, we demonstrated that the long-term VMT could enhance motor skill learning in a similar motor learning paradigm, rotorod. This task provides a quantitative analysis of motor learning, in contrast to wheeling. The enhanced motor learning in B6/CBA F1 mice after long-term VMT presents direct evidence showing that long-term VMT is able to trigger the “use-dependent plasticity”. Most importantly, we have demonstrated that during the development of this use-dependent plasticity, the expression of the NR2B transgene significantly facilitates the effect of motor training, which established the first GEI model in motor learning.

How the recombinant NMDAR facilitates this use-dependent plasticity in the cerebellum is not clear from the current study. Since the long-term modification of neurotransmission efficacy at synapses either potentiation (LTP) or depression (LTD) is considered as a cellular basis for learning behaviors [57], it might be possible that the expression of the NR2B transgene or/and motor training together change these “synaptic behaviors”. Especially, parallel fiber LTD in the cerebellum may be particularly important [47], because this type of synaptic plasticity is typically associative, since neither stimulating parallel fiber alone nor climbing fiber alone can produce the depression [58], and activity-dependent, as it is induced by repetitive conjunctive stimulation [59]. Indeed, there is robust evidence showing that parallel fiber LTD is associated with motor learning [14], [60], [61]. However, at the same time, controversial findings were also reported. For example, it has been found that mice with diminished parallel fiber LTD, either by genetic deletion [14] or pharmacological blockage [62] have normal motor learning, suggesting that a particular plasticity mechanism may not support all cerebellar learning or/and that motor learning is controlled by multiple plasticity mechanism [63], [64]. Indeed, there are at least four forms of LTD and three forms of LTP in the cerebellum. The significance and interaction among them are complicated. Therefore, this study did not ambitiously explore the potential implication of these synaptic mechanisms in this specific GEI, as weak-point in this study.

Finally, at the system level again, we demonstrated that this GEI is both developmental stage-dependent and task-specific. The effect was observed only when the motor training was conducted at the stage that is critical for some specific motor skill development [31]. Since from that time on, the transgene has already been fully expressed, this developmental stage-dependent feature highlights a development-related role of the recombinant NMDAR with NR2B in replace of NR2C in the development of motor skill learning. Very interestingly, a recent study in adult neurogenesis revealed a critical developmental stage existence, during which synaptic plasticity is enhanced because of the higher NR2B expression level [39]. Therefore, the recombinant NMDAR with NR2B may underlie this developmental stage-dependent effect. This developmental stage-dependent effect is also well observed in human studies [26], [32]. At the same time, it has been well-established that the development of motor skills is task-dependent, that is a specific training in a task is required for the development of a specific motor skill [21], [22], [65]. It is daily experience that training in a water pool, for example, improves one's swimming skill but not essentially change one's skill in bicycling. Our studies also reproduced this specificity in the animals. However, it should be noted that evidence is available that the cerebellum is also involved in spatial navigation [66]. In our study, however, we found that neither NR2B expression in the cerebellum nor GEI with wheeling changed the navigation behavior (Figure 8). This discrepancy suggests that as a motor-controlling organ, the cerebellum is required for normal navigation so that the lesion to the cerebellum may lead to deficits in spatial learning. However, the changes in the cerebellum that are able to enhance, but not impair, motor learning may not sufficient enough to enhance navigation strategy, which is essentially controlled by the medial temporal lobe including the hippocampus [1], and therefore, spatial learning and memory is unaffected in our transgenic mice.

In conclusion, our results for the first time validated a GEI model in motor learning and further indicated that the expression of gene(s) that are involved in regulating synaptic plasticity in the cerebellar granule cells may constitute a molecular basis for GEI in motor skill learning.

## Methods

### Generation of cerebellar granule cell-specific NR2B transgenic mice

As shown in Figure 1A, the tTA/tetO inducible gene manipulation system was used to produce these mice. Two constructs were made. GABA-α6-tTA construct consisted of a 12 kb of GABA-α6 promoter, an IRES element (615 bp, pIRES, Clontech), tTA (1 kb, pTte-Off, Clontech), and SV-40 poly-A signal (0.8 kb, p265). The transgenic cassette was released by Mlu I and Xho I from the backbone. The tetO-tau-Lac-Z construct consisted of tetO mini promoter (0.4 kb, pTRE2, Clontech), an artificial intron (0.3 kb, p265), tau-Lac-Z (5.5 kb, in pSK (+), (which was kindly provided by Dr. John Thomas at the Salk Institute, San Diego), and the SV-40 poly-A. The transgenic cassette was released by Sal I. The tetO-NR2B construct consisted of the tetO mini promoter, the artificial intron, mouse NR2B cDNA, and the SV-40 poly-A. The transgenic cassette was released by Sal I. After being linearized with the enzymes as indicated, the transgene cassettes were respectively injected into pro-nuclei of B6/CBA F1 zygotes as described elsewhere [67]. The gene copy number in founder mice was determined by Southern blot. In order to have a medium expression level of the transgene, founders with transgene copy number at about 3–5 were used to breed double transgenic mice. As both zygote donors and mice used for founder segregation (or single transgenic mouse production) were all B6/CBA F1 mice, the overall genetic background for all the littermates were the same. The genotypes after the F1 generation were determined by PCR analysis of tail DNA with primers respectively for tTA transgene and NR2B (SV-40) transgene.

### Molecular characterization of tg mice

We used *in situ* hybridization with a ^35^S-labeled oligo probe that could recognize the NR2B transgene only to detect the expression pattern. The procedures were described in our previous publication [38]. Western blot was used to determine the expression level and the procedures were the same as our previous publication [38]. Briefly, total of 50 µg of protein from the cerebella of either control (cont) or tg mice were separated on 7.5% SDS-PAGE and the PVDF membranes (Immobilon-P membranes, Millipore, Bedford) were incubated with anti-NR2B antibody (Chemicon) and anti-β-actin antibody (Sigma), respectively. The blotting signal was visualized with an ECL detection system (Pierce). Re-probe to anti-β-actin antibody was used to normalize the protein-loading amount. Densitometry was performed with using Image-J software (version 1.39c) to determine the expression level.

### EM immunocytochemistry

The procedures were similar to or previous publication [68]. Briefly, after fixation, coronal blocks (0.5 mm thick) of cerebella from both control (n = 5) and tg mice (n = 5; 2 months old, all at 2 months old) were made with a Vibratome (Leica) and then were processed for freeze-substitution and low-temperature embedding. Ultra-thin sections were cut and mounted on nickel grids (Electron Microscopy Sciences). Post-embedding immunogold labelling was performed with rabbit polyclonal anti-NR2B antibody (Novus; 5 µg/ml) overnight followed by anti-rabbit IgG F-10 nm gold-tagged antibody (Electron Microscopy Sciences, 1∶40). In all experiments, related immunogold particles were absent, indicating the specificity of the specificity of the procedures. Ultra-structural analyses were performed on a Jeol 1200EX electron microscope (Tokyo, Japan), and imaged with a CCD camera (Advanced Microscopy Techniques, MA). The identification criteria for MF/granule cell synapse were the same as described elsewhere [69]. The EM images were imported into Adobe Photoshop 8.0, where they were sized, labelled and optimized data analysis. The axospinous and axodendritic synapses throughout the cerebellum appeared morphologically normal in wt and tg mice. A synapse was considered positively immunolabeled for NR2B when at least two gold particles were observed within the spine cytoplasma, post-synaptic density (PSD), or synaptic cleft.

### Electrophysiological recordings

Whole-cell patch-clamp recording of NMDAR responses were recorded in cultured cerebellar granule cells as described previously [70]. Briefly, cerebellar granule cells were respectively cultured from control and tg pups at postnatal 8 (P8) days. The NMDA currents were measure at Div 13–18 days with whole-cell patch-clamp recordings. NMDA (300 µM) for 0.5 seconds (sec) with 10 µM glycine in Mg^2+^-free solution was used to trigger NMDA-current in both control and tg cells. Peak and steady-state currents evoked during 0.5 sec agonist applications were determined from means of 3 current traces. Peak NMDA-evoked responses were divided by peak responses to kainate for normalization. Fractional block by ifenprodil was calculated comparing the peak NMDA-evoked response in the presence of ifenprodil to the mean of those evoked before and after application of ifenprodil.

### Ca^2+^ imaging in cultured cerebellar slices

The procedures were described elsewhere [71]. Briefly, transverse cerebellar slices (350 µm) were made at 4°C from brains of 30-day-old mice and maintained in artificial CSF 23°C. Slices were loaded with Ca^2+^-sensitive fluorescent dye (Fluo-4 AM, calcium indicator 10 µM, Molecular Probes) and were examined on the stage of an inverted microscope (Olympus IX81; Olympus Optical, Tokyo) equipped with a DSU spinning disk confocal system and a back-thinned EM-CCD camera (Hamamatsu). Imaging was visualized through a 40×water-immersion objective with the direct application direct applications of NMDA (0.1 mM, Sigma) and glycine (10 µM, Sigma) into the perfusion buffer. Images were collected at 0.2–3 Hz and the analysis was performed offline, using as region of interest (ROI) of the entire soma of individual neurons. Ca^2+^ influx was analyzed by randomly selecting a region that at least contains 10 neurons from each slice (5 mice in each group) with blind to the genotypes.

### Morphological characterizations

For both Nissl and immunostaining, saggital brain sections from both control and tg mice at 3 months old with mixed sexes were prepared. The procedures were the same as our previous publications [72]. For EM, samples were prepared, as described above, from cerebella of both control and tg mice at 3 months old. We used a random sampling method to select sections. In order to have three systematic random sampling fields from each cerebellum, six coronal blocks (0.5 mm thick) were prepared from each mouse with each two blocks from each lob of the cerebellum. After sectioning, at most 60 electron micrographs at ×12,000–16,000 magnification from each block was randomly taken with the requirement that each micrograph must contain at least one glomerulus (mossy fiber synapse). The experimenters were blind to genotype of each individual mouse. The identification criteria for mossy fiber synapses were the same as described above [69]. Only synapses with clearly visible postsynaptic membrane and PSD were selected for quantitative analysis. The number of synapses in each glomerulus was counted and averaged from 360 micrographs from each mouse. There were 4 mice in each group with mixed sexes. It should be noted that in glomeruli there are synapses between granule cell dendrites and Golgi cell axons. As these types of synapses are inhibitory synapses, they can be distinguished morphologically from mossy fiber synapses as described previously [73].

### Motor learning in rotorod task

The procedures were similar to publication elsewhere [74]. Briefly, two versions of tests were used. In fixed-speed rotorod test, the speed of the rod was fixed and two speeds, 20 rpm and 25 rpm, respectively. Mice were first examined at the first speed (20 rpm) up to the completion of the test and then were examined at the second speed (25 rpm). Mice were given three trials every day and each trial was ended by either falling down from the rod or staying on it over 100 seconds. Any mice that were able to stay on the running rod for 100 seconds in three consecutive trials were considered that these mice had learned the task (learned criterion). The final measurement was the total time from every trial that was required for the mouse to reach the learned criterion, but did not include the final 3×100 seconds. In accelerated-speed rotorod test, mice were subjected to three trials per day (session) in 3 consecutive days. Each trial lasted for 5 minutes. The starting speed of the rod was 5 rpm and the speed gradually increased following time in each trial at a constant rate of 0.2 rps so that the speed would be up to 65 rpm at the end of each 5-minut trial. The time for mouse keeping on the rod was recorded and the average time from three trials were calculated in each training session.

### VMT

After weaning (P20), each 10–12 mice from both control and tg mice (male) were transferred into a bigger cage (1×0.75×0.75 m high), in which two wheels were presented. Except for this, the other conditions were the same as in their standard home cages. The other housing environment such as the light/dark cycle etc was also the same. Mice were allowed to run the wheels freely for 60 days and then subjected to task examination. It should be noted that this training paradigm was not a typical environmental enrichment in the rodents.

### Fear-conditioning test

A fear-conditioning working-station (Coulbourn Instruments, Allentown, PA) and one-trial protocol were used as our previous publication [38]. Briefly, during training session, mice were individually put into the shock chamber and were allowed to freely explore the environment for 150 seconds. Immediately after this, a tone at 90 dB and 2,800 Hz (CS) was delivered for 30 seconds and at the last 2 seconds a foot shock at 0.8 mA was delivered to the mice for 2 seconds (US). After the pairing of CS/US, mice were allowed to stay in the chamber for another 30 seconds and then were returned to their homecages. A retention test was conducted 24 hours after the training. For contextual conditioning, mice were individually put back into the chamber where they received the shock and the freezing response was recorded for 4 minutes with a sampling method at an interval of 5 seconds. For cued conditioning, mice were individually put into a novel chamber for 3 minutes and then the same tone used during the training session was delivered for another 3 minutes. The freezing response was recorded in both pre-tone and during-tone periods.

### Morris water-maze test

Spatial learning and memory was examined with a Morris water-maze as described in our previous publication [38]. Briefly, a circle water tank (diameter 100 cm and 75 cm in high) was filled with water and the water was made opaque with non-toxic white paint (Reeves &Poole group, Toronto, Canada). A black curtain with three visible signs on it was surrounded the swimming pool about 1 meter apart from the water tank. A round platform (diameter 15 cm) was hidden 1 cm beneath the surface of the water at the center of a given quadrant of the water tank. Training was carried out for continuous 8 days (8 sessions) and each session consisted of 4 trials. For each trial, mouse was released from the wall of the tank by face against to the wall into the water and was allowed to search, find, and stand on the platform for 10 seconds within the 60-second trial period. An interval of 2 hours was set between each two trials in each animal. For each training session, the starting quadrant and sequence of the four quadrants from where mouse was released into the water tank was randomly chosen so that it was different among different sessions in each animal and was different between different animals. The navigation of mice was recoded by a video-camera and the task performances including swimming paths, speed, and time spent in each quadrant were recorded by using an EthoVision video tracking system (Noldus). A probe test was conducted on 24 hours after the completion of the training. During the probe test, the platform was removed from the pool, and the task performances were recorded for 1 minute. The time spent in each quadrant was analyzed.

### Statistical analyses

Data were analyzed by Student's *t* test, Mann-Whitney *U* test, and one-way ANOVA followed by *post hoc* analysis of Fisher's PLSD test, where appropriate. A p value less than 0.05 was considered significance.
